# Large-Cell Esophageal Neuroendocrine Tumor Leading to Hepatorenal Syndrome

**DOI:** 10.7759/cureus.23720

**Published:** 2022-04-01

**Authors:** Zaryab Umar, Usman Ilyas, Ibironke Otusile, Ian Landry

**Affiliations:** 1 Internal Medicine, Icahn School of Medicine at Mount Sinai, Queens Hospital Center, New York City, USA; 2 Medicine, Icahn School of Medicine at Mount Sinai, New York City Health and Hospitals/Queens, New York City, USA

**Keywords:** esophageal cancer, hepatorenal syndrome, neuroendocrine neoplasm, neuroendocrine carcinoma of esophagus, large cell neuroendocrine carcinoma

## Abstract

Neuroendocrine tumors are tumors that arise from the enterochromaffin cells in the neuroendocrine tissue found throughout the body, particularly the digestive tract, pancreas, and thymus. Neuroendocrine tumors of the esophagus are extremely rare and highly aggressive in nature. We present the case of a 55-year-old Hispanic male who initially presented to the emergency department with right-sided abdominal pain. Imaging revealed innumerable lesions occupying half of the liver parenchyma. Subsequent endoscopy with biopsy of the esophageal and liver lesions along with immunohistochemistry staining was suggestive of a large cell neuroendocrine tumor. He later presented with generalized weakness and right-sided abdominal pain with worsening hepatic and renal function. Over the course of the patient's stay in the hospital, his mental status progressively deteriorated. Given the deranged hepatic and renal function, chemotherapy could not be initiated. The patient's family decided against hemodialysis considering his poor prognosis and the patient expired on day 15 of admission. The case report highlights the aggressiveness of one of the rare esophageal malignancies. It is crucial to establish diagnosis at the earlier stages of the disease with prompt treatment in order to avoid serious complications such as hepatorenal syndrome, which resulted in rapid deterioration of our patient's clinical status. More research is necessary in order to establish guidelines to treat neuroendocrine tumors of the esophagus.

## Introduction

Esophageal malignancies are rare and arise in approximately five out of 100,000 patients. The most common types include squamous cell carcinoma and adenocarcinoma [1]. Esophageal neuroendocrine neoplasms (E-NENs) are rarer than other gastro-entero-pancreatic neuroendocrine tumors (NETs), accounting for 0.03-0.05% of all esophageal malignancies, with more than 4000 cases described in the literature [2]. Esophageal neuroendocrine tumors (NETs) and esophageal neuroendocrine carcinomas (E-NECs) are types of E-NENs. E-NECs may be further classified into two morphological types; small cell and large cell type, with the former being more common in frequency [2-5]. They are more common in males with a mean age of presentation of 65 years [2]. Dysphagia, weight loss, odynophagia, retrosternal or epigastric pain, hoarseness, reflux disease, hiccups, and bleeding are the most common initial presentations [6-8]. Lymph nodes are the most common sites of metastasis with distant metastases seen in the liver, lung, and bone [2,6]. The majority of E-NECs characteristically have a poor prognosis with aggressive behavior and early dissemination [2,4,6,9]. Due to its rarity, there are no specific guidelines on management. Treatment depends on the type, grade, and stage of the disease with a multidisciplinary approach using surgery, chemotherapy, and radiotherapy [6,7]. We present a case of a large cell neuroendocrine tumor of the esophagus, which presented initially as right-sided abdominal pain and discomfort with metastasis to the liver, leading to hepatic and renal dysfunction likely due to a hepatorenal syndrome, and eventual death of the patient.

## Case presentation

**Figure 1:**
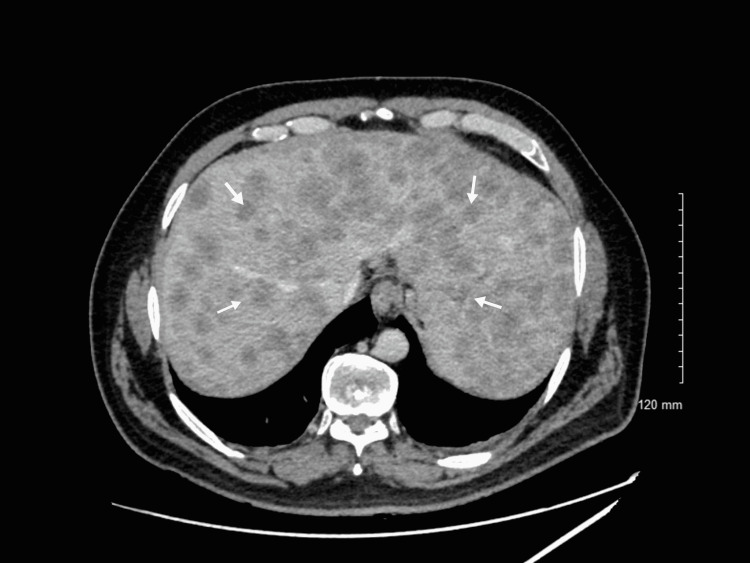
CT scan of the abdomen and pelvis revealing enlarged liver with innumerable low-density lesions occupying half of the liver parenchyma (white arrows).

**Figure 2:**
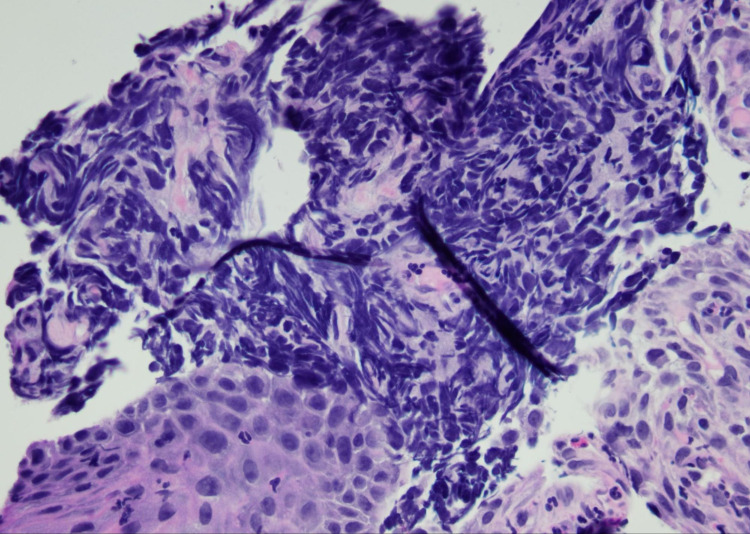
Esophageal biopsy showing high-grade neuroendocrine carcinoma.

**Figure 3:**
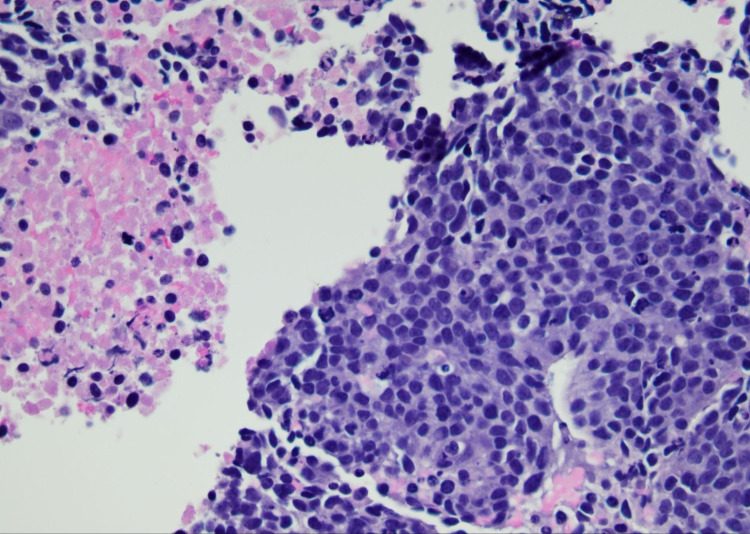
Liver biopsy showing metastatic high-grade neuroendocrine carcinoma with necrosis

A 55-year-old Hispanic male presented to the emergency department with right-sided abdominal pain for two weeks duration that was initially intermittent but then progressed to being constant with 10/10 in severity. Laboratory assessment revealed total bilirubin of 10.8 mg/dL with direct bilirubin of 8.6 mg/dL. Liver enzymes were deranged with aspartate aminotransferase (AST) of 303 U/L, alanine transaminase (ALT) of 203 U/L, and alkaline phosphatase of 260 U/L. Gamma-glutamyl transferase (GGT) was elevated to 1256 U/L. Carcinoembryonic antigen (CEA) and cancer antigen 19-9 (CA 19-9) were elevated to 149 ng/mL and 239 U/mL, respectively (Table 1). CT scan of the abdomen and pelvis revealed enlarged liver with innumerable low-density lesions occupying half of the liver parenchyma (Figure 1). Given the high suspicion of malignancy, the patient underwent endoscopy revealing minute foci of carcinoma with ulcer and subsequent esophageal and liver biopsies. Biopsy findings were suggestive of high-grade metastatic neuroendocrine tumors (Figure 2 and Figure 3). Immunohistochemistry studies done on the esophageal and liver biopsies were positive for p63, synaptophysin, anti-cytokeratin (CAM 5.2), AE1/AE3, thyroid transcription factor 1 (TTF-1), focally positive for chromogranin, neuron-specific enolase (NSE), cytokeratin (CK) 7, and negative for CK 5/6. Proliferative index marker Ki-67 labeled approximately 90% of the tumor cells.* *The patient was diagnosed with stage 4 neuroendocrine tumor of the esophagus with metastasis to the liver. He was discharged with an outpatient referral to hematology/oncology for further management including chemotherapy.

**Table 1:** Lab values at the time of patient's first presentation compared to the most recent admission AST: aspartate aminotransferase; ALT: alanine transaminase; CEA: carcinoembryonic antigen; CA: cancer antigen; GGT: gamma-glutamyl transferase; BUN: blood urea nitrogen

Laboratory parameter (reference range with units)	Values at the time of patient's first presentation vs most recent admission
Albumin (3.5-5.2 g/dL)	3.1 vs 2.7
Total Bilirubin (0.0-1.2 mg/dL)	10.8 vs 32.6
Direct Bilirubin (0.0-0.3 mg/dL)	8.6 vs >20.0
Alkaline phosphatase (40-129 U/L)	260 vs 587
AST (5-40 U/L)	303 vs 638
ALT (0-41 U/L)	203 vs 198
GGT (8.0-61.0 U/L)	1256 vs 1368
CEA (0.0-3.8 ng/mL)	149
CA 19-9 (<=35 U/mL)	239
BUN (6-23 mg/dL)	19 vs 83
Creatinine (6-23 mg/dL)	1.11 vs 3.39

**Figure 4:**
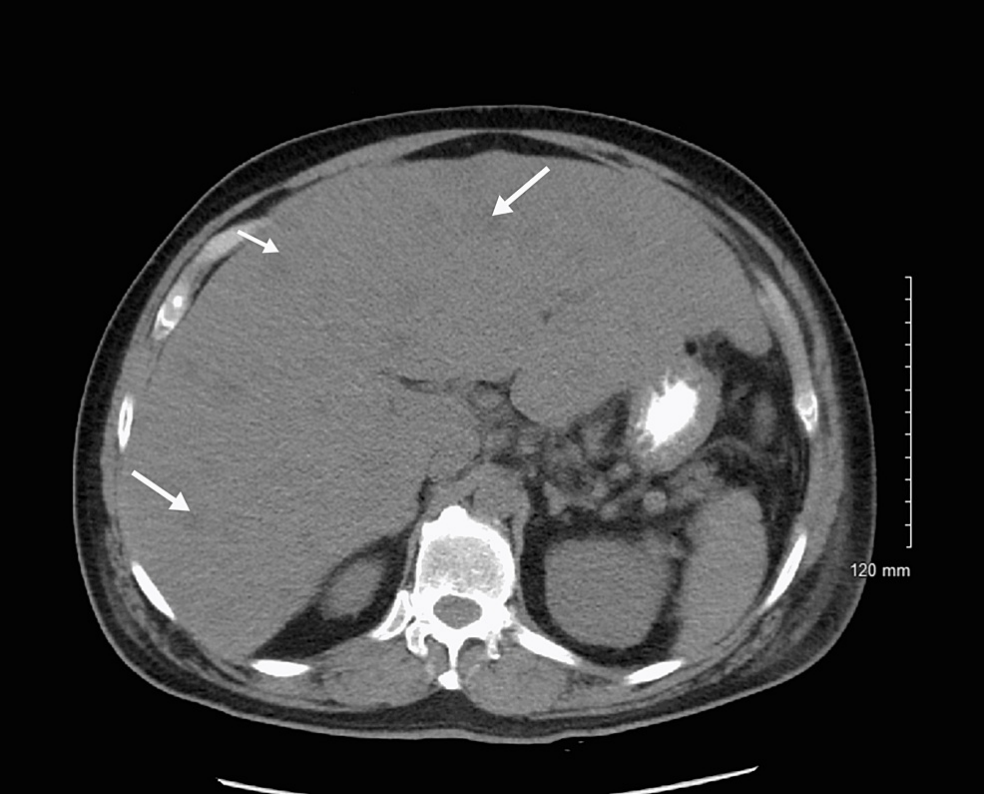
CT abdomen and pelvis showing hepatomegaly with numerous metastasis (white arrows).

The patient returned two weeks later to the emergency department complaining of generalized weakness and right-sided abdominal pain, rated as 8 out of 10. The patient's symptoms started as subjective weight loss over the course of two weeks, which progressively developed into loss of appetite, generalized weakness, and dyspnea upon rest and exertion. His vital signs were stable with blood pressure (BP) of 135/74, pulse of 85 beats per minute, temperature of 97.8F (36.6C) (Oral), respiratory rate of 18 cycles/minute, and oxygen saturation (SpO2) of 96% on ambient air. The patient was alert and oriented to person, place, and time with no focal deficit present. Hepatic and renal function studies were suggestive of worsening liver and kidney function compared to the patient's prior presentation when he was diagnosed with a neuroendocrine tumor of the esophagus (Table 1). Non-contrast CT scan of the abdomen and pelvis revealed hepatomegaly with numerous metastases (Figure 4).

On the second day of hospitalization, the patient’s mental status changed and he was more lethargic and no longer oriented to time. A nephrology consult was placed for worsening renal function with anion gap metabolic acidosis. Nephrology recommended the insertion of a foley catheter and a bicarbonate drip. 

On day four, the patient was upgraded to the step-down unit for closer monitoring due to his deteriorating condition. In the step-down unit, the patient was initially lethargic and arousable only to physical stimuli. At this time, the family decided on Do-Not-Resuscitate/Do-Not-Intubate (DNR/DNI) code status. He was treated with lactulose and bisacodyl with improvement in mental status and oral intake. Rifaximin was started and his bicarbonate drip requirements were reduced. The patient was stabilized and transferred back to the medicine floor. Because of the patient’s deranged renal and hepatic function, chemotherapy could not be initiated and nephrotoxic and hepatotoxic drugs were avoided.

On day nine, he developed a fever of 103F and responded only to painful stimuli. Due to his deranged hepatic function, his fever was controlled only with ice packs and cooling blankets. A nephrology follow-up suggested that his rapid progression of renal dysfunction, hepatic encephalopathy, and metabolic acidosis (Table 2) likely correlated with a type 1 hepatorenal syndrome. Because of his terminal stage, a group decision with the family was made to forgo hemodialysis, and he expired on day 15 of his admission.

**Table 2:** Patient's hepatic and renal function as well as other pertinent labs throughout the patient's most recent stay at the hospital AST: aspartate aminotransferase; ALT: alanine transaminase; BUN: blood urea nitrogen

Lab with reference range and units	Day 2 of admission	Day 4 of admission	Day 9 of admission	Day 10 of admission
Albumin (3.5-5.2 g/dL)	2.7	3.3	2.8	3.0
Total Bilirubin (0.0-1.2 mg/dL)	32.6	33.8	34.9	34.9
Direct Bilirubin (0.0-0.3 mg/dL)	>20.0	>20.0	>20.0	>20.0
Alkaline phosphatase (40-129 U/L)	587	474	254	217
AST (5-40 U/L)	638	787	577	393
ALT (0-41 U/L)	198	193	180	136
BUN (6-23 mg/dL)	83	115	205	201
Creatinine (0.70-1.20 mg/dL)	3.39	6.67	7.69	7.77
Sodium (136-145 mmol/L)	130	135	152	151
Potassium (3.5-5.1 mmol/L)	5.8	3.9	4.6	4.8
Chloride (98-108 mmol/L)	95	86	98	98
Bicarbonate (22-29 mmol/L)	15	25	26	27
Anion gap (8-16 mEq/L)	20	24	28	26

## Discussion

Esophageal neuroendocrine neoplasms (E-NENs) consist of well-differentiated E-NETs, poorly differentiated E-NECs, and mixed neuroendocrine-non neuroendocrine neoplasms (MiNENs) [2]. E-NECs are further classified into two morphological subtypes: (1) small cell and (2) large cell type, with the former being much more common (90%) [2-5]. One theory, the stem cell theory, suggests that these malignant cells arise from either pluripotent stem cells or neuroendocrine cells present throughout the body, including thymus, pituitary, and adrenal glands, lung, pancreas, and the gastrointestinal tract (Merkel cells) [3,7,10,11]. In our patient, the stem cell theory is supported as the tumor cells are immunoreactive for p63, a known marker for stem cells, and negative for CK20, a marker for Merkel cells, the neuroendocrine cells of the gastrointestinal tract. Additionally, squamous and glandular differentiation and coexistence are common, supporting multidirectional differentiation of the primary source [10].

According to the WHO’s classification, the site of origin of the NET is related to its clinicopathological behavior and all NETs should be considered potentially malignant [6]. E-NENs are the rarest of the gastro-entero-pancreatic NETs, accounting for 0.03-0.05% of all esophageal malignancies, with only 4,000 cases described in the literature [2]. In fact, a large American study of over 13,000 cases of NETs diagnosed between 1975 to 1999 concluded that 67% of these were located in the digestive tract. Of these, 8% originated from the small intestine and less than 1% were from the esophagus [4,12,13]. The majority of E-NECs have a poor prognosis with aggressive behavior and early dissemination [2,4,6,9]. As a consequence of the rarity of this neoplasm, no definitive guidelines on its management were previously available. However, new data is now emerging as more cases are now diagnosed with enhanced techniques, resulting in an increase in tumor incidence [2,6].

The mean age of diagnosis for patients with E-NECs is 65, with a male to female predominance ratio of 6:1 [2]. The majority of E-NENs occur in the lower third of the esophagus [2,6,7]. The majority of these cases are nonfunctional with tumors causing a mass effect to produce symptoms such as dysphagia, weight loss, odynophagia, retrosternal or epigastric pain, hoarseness, reflux disease, hiccups, and bleeding [6-8]. Hoarseness may occur as a result of recurrent laryngeal nerve paralysis [6]. Rarely, functional tumors may occur with symptoms pertaining to the type of hormone or peptide produced by the neoplasm. Very rarely, E-NECs are diagnosed incidentally on endoscopic examination in asymptomatic patients. The most common sites of metastatic lesions are locoregional lymph nodes and distant metastases to the liver, lung, and bone; however, brain metastasis is rare [6,7].

Endoscopy with biopsy is the primary tool used for diagnosis, with imaging techniques such as CT reserved for staging the neoplasm [6,7]. Macroscopically, neuroendocrine carcinomas exhibit prominent submucosal growth, usually covered by normal epithelium but may have ulceration at the center [3,6].

Identifying and differentiating light microscopic features of large cell neuroendocrine tumors (LCNET) from esophageal squamous cell carcinoma, adenocarcinoma is the most difficult diagnostic factor, since rosette-like structures, the best marker for recognition of neuroendocrine differentiation, can be confused with glandular formations, thereby erroneously misdiagnosing as poorly differentiated adenocarcinoma [8,10,14]. Similarly, more solid areas of the neoplasm may display squamoid morphologic findings, resulting in the diagnosis of poorly differentiated squamous cell carcinoma [10,14]. This misdiagnosis occurred in our case before the histology was reviewed and labeled correctly as LCNET [10,15].

Neuroendocrine differentiation is also demonstrated by immunohistochemical stains including synaptophysin, chromogranin A, neuron-specific enolase, and CD56, with positivity ranging from 60% to 100% [2,6,10,13]. Additionally, a Ki 67 or mitotic index of 20% or more is required for diagnosing neuroendocrine carcinoma, whereby the tumors with Ki 67 positivity of below 20% are diagnosed as neuroendocrine tumors [3,6,15]. Similarly, Alcian blu-PAS and CK 7 for adenocarcinoma and p63, p40, and CK 5/6 for squamous cell carcinoma identification can be used [2,16].

After initial diagnosis, further evaluation and staging are done with a contrast-enhanced multiphase CT scan, with 18 F-fluoro-deoxy-glucose (FDG) positron emission tomography (PET)/CT recommended for staging and detecting recurrence. Endoscopic ultrasound remains the most useful modality for detecting depth of invasion as well as lymph node involvement [6,7].

According to the Veterans Administration Lung Study Group (VALSG) classification, the tumor is classified either as a limited or extensive disease. Limited disease is defined as disease confined to the esophagus and adjacent organs with or without local lymph node involvement. Extensive disease occurs when the tumor extends beyond regional boundaries [7].

Treatment is dependent on the type (small or large cell), grade, and stage (limited disease, extensive disease, or tumor, nodes, and metastases (TNM) classification) [6,10]. Due to its rarity, no specific therapeutic guidelines have been established but a multidisciplinary approach with surgery, chemotherapy, and radiotherapy is recommended [6,7]. The highest survival rates have been observed with a multimodal approach [2,7]. Chemotherapy is the first-line treatment as disease is often disseminated at the time of diagnosis. Additionally, chemotherapy assists with the prevention of recurrent metastatic lesions [6,7]. Platinum-based chemotherapy regimens (cisplatin/etoposide and cisplatin/irinotecan) are typically used given the histological similarity to small cell lung cancer [6,7,17]. According to Yan et al., levels of NSE may be used as a prognostic factor in response to chemotherapy regimen of cisplatin and etoposide as patients with NSE levels below 17 respond more favorably to treatment [6]. Surgery has a limited role in the management of NETs given poor postoperative quality of life, high perioperative morbidity and mortality, and little survival benefit [2,6,7,14]. Minimally invasive transhiatal surgery is advised for early cancers of middle and lower esophagus, as well as patients unable to undergo thoracotomy. Although transthoracic esophagectomy is also widely used, it is not considered high-yield. Compared to chemotherapy alone (24 months), radiation therapy only offers only months of survival benefit; therefore, it is used in conjunction with other treatment modalities [6,7].

Age, disease extent, TNM classification, and type of treatment (local +/- systemic) are the main prognostic factors affecting survival [2]. The median survival rate is 4-16 months, reported in studies with both advanced and limited stage NET. However, survival rate cannot be accurately established because it is difficult to determine from literature if the survival time was calculated from time from diagnosis or time from initiation of treatment [7].

## Conclusions

This case report sheds light on one of the rare esophageal malignancies and its initial presentation. It also highlights the aggressiveness of the disease with metastasis to the liver that can lead to rapid hepatic and renal dysfunction with eventual death. Endoscopy with biopsy, imaging modalities for staging, and immunohistochemical markers for the diagnosis of the disease have been discussed. To the best of our knowledge, no specific guidelines are available for the treatment of neuroendocrine tumors of the esophagus and further research needs to be conducted to establish guidelines regarding its management.
